# Intensity-dependent effect of treadmill running on lubricin metabolism of rat articular cartilage

**DOI:** 10.1186/ar4101

**Published:** 2012-11-24

**Authors:** Guo-Xin Ni, Lei Lei, Yue-Zhu Zhou

**Affiliations:** 1 Department of Orthopaedics and Traumatology, Nanfang Hospital, Southern Medical University, 1838 Guangzhou Avenue (N), Guangzhou 510515, China; 2 Department of Rehabilitation Medicine, Longyan People's Hospital, 31 Denggao Road (W), Longyan 364000, China; 3 Department of Rehabilitation, 1st Affiliated Hospital, Fujian Medical University, 20 Chazhong Road, Fuzhou 350005, China

## Abstract

**Introduction:**

We aimed to understand the changes in cartilage lubricin expression and immunolocalisation in responsed to treadmill running with different intensities in a rat model.

**Methods:**

A total of 24 male Wistar rats were randomly assigned into groups of control (CON), low-intensity running (LIR), moderate-intensity running (MIR), and high-intensity running (HIR). Rats in LIR, MIR, and HIR groups were trained for 8 weeks on the treadmill with low, moderate, and high intensity, respectively. After sacrifice, femoral condyles were collected to take histological observation for cartilage characteristics, and immunohistochemistry for lubricin. In addition, cartilage samples were obtained to assess *PRG4 *and *TGF*-β mRNA expression by quantitative RT-PCR.

**Results:**

Histological examination showed osteoarthritic changes in rats after eight weeks of high intensity running. In comparison to CON group, significantly lower Mankin score was found in LIR and MIR groups, whereas, HIR group had significantly higher Mankin score than either CON, LIR, or MIR group. On the other hand, both LIR and MIR groups have significantly higher lubricin content than CON group, whereas, significantly lower lubricin content was found in HIR group compared with CON, LIR or MIR group. A significant inverse correlation was detected between the lubricin content and Mankin score. In addition, considerably higher mRNA gene expression of *PRG4 *and *TGF*-β was found in LIR and MIR groups, compared with those in CON and HIR groups.

**Conclusions:**

There is a marked intensity-specific effect of running on the immunolocalisation and gene expression of lubricin in cartilage, which is inversely correlated with Mankin score. Our findings provide evidences that mechanical factors are key determinants of lubricin metabolism *in vivo*.

## Introduction

In addition to transmitting load, another major function of articular cartilage is to provide a low-friction surface that allows the bones of diarthrodial joints to slide smoothly against each other. Such remarkable frictional properties of the tissue are achieved, at least in part, by lubricin, a mucinous glycoprotein synthesized and secreted into synovial fluid both by chondrocytes in the superficial zone of articular cartilage [1] and by synoviocytes [2], and which is encoded by the proteoglycan 4 (*PRG4*) gene [3]. There are a number of post translational products of the *PRG4 *gene [2-6], including megakaryocyte stimulating factor precursor and superficial zone protein (SZP). In this article, these homologous proteins are referred to as lubricin.

While functioning as a boundary lubricant in articular joints, lubricin is also recognized to have a major protective role in preventing cartilage wear, and synovial cell adhesion and proliferation [7]. Obviously, lubricin plays an important role in articular joint physiology, and the loss of accumulation of lubricin may have a role in the pathology of osteoarthritis (OA). Indeed, lubricin synthesis is down-regulated in a number of animal models of OA [8,9]. In addition, in mice lacking the lubricin gene, there is alteration of the articular surface and attendant degradation of articular cartilage, causing early-onset noninflammatory joint damage and failure [10]. Furthermore, a recent study by Flannery *et al. *[11] demonstrated that intra-articular lubricin injection following an anterior cruciate ligament (ACL) injury was beneficial in retarding the degeneration of cartilage and the development of post-traumatic OA.

*In vitro *evidences suggest that mechanical stimulation can affect the biosynthesis and secretion of lubricin. Nugent *et al. *[12,13] found that both static and dynamic shear stimulation applied to cartilage explants altered cartilage secretion of lubricin relative to that of unloaded controls. More recently, using a bioreactor, Nugent-Derfus *et al. *[14] suggested that lubricin secretion rate varied markedly over the joint surface following 24 hours of continuous passive motion (CPM). Nevertheless, as the load experienced by natural joints is more complicated than simple compression, shear or pressurization, it is difficult to extrapolate these *in vitro *results to the *in vivo *situation.

Running is one of the most common weight-bearing activities. However, the *in vivo *regulatory effect of running load on the biosynthesis of lubricin remains unknown. For one thing, marked magnitude-dependent regulatory effects of mechanical stimuli have been suggested *in vitro *on cartilage biosynthesis of lubricin molecules [12,13]. On the other hand, the homeostasis of cartilage was stabilized in a physiological range of mechanical load, non-physiological mechanical load, both overload and reduced load, may have deleterious effects [15-17]. Specifically, moderate running exercise was found to protect against cartilage degradation in hamsters that had spontaneously developed OA [18]. However, excessive running load has been correlated with deleterious effects on knee cartilage in rats that did not spontaneously suffer from OA [19-21]. A recent study reported that there was a significant inverse correlation between the lubricin concentration in synovial fluid and the Mankin score of guinea pig knee cartilage [22]. It is therefore hypothesized that the running-induced load may regulate *in vivo *biosynthesis of lubricin with intensity dependence. To assess this hypothesis, the current study was undertaken to determine the changes in cartilage lubricin expression and immunolocalisation in response to treadmill running with different intensities on the knee joint of rats.

## Materials and methods

### Experimental animals and exercise protocols

A total of 24 male Wistar rats (12 to 13 week old, weight 200 to 250 g) were randomly and evenly assigned to one of four groups as follows: 1) sedentary control (CON, *n *= 6); 2) low intensity running (LIR, *n *= 6); 3) moderate intensity running (MIR, *n *= 6); and 4) high intensity running (HIR, *n *= 6). Rats were housed in cages under controlled light/dark (12/12 h) and temperature (22 ± 1°C) conditions, and were provided with food and water *ad libitum*. They were adapted to laboratory conditions for 1 week before experiments began. This study was approved by the animal ethics committee of the institute.

All rats were firstly accustomed to exercise for 1 week, by running on a treadmill at a speed of 10 m/min for 30 minutes/day. Subsequently, animals in the LIR, MIR, and HIR groups were regularly trained according to the running protocol (Table 1) for 8 weeks, to elicit the low, moderate, and high intensity exercise respectively, for the Wistar rats, [23], while the CON group served as the control. All experiments were conducted in accordance with our institutional guidelines for the care and use of experimental animals.

**Table 1:** Treadmill running protocols for rats

Group	Speed	Inclination	Duration	Frequency
LIR	15.2 m/minute	0°	60 minutes	5 days/week
MIR	19.3 m/minute	5°	60 minutes	5 days/week
HIR	26.8 m/minute	10°	60 minutes	5 days/week

LIR, MIR and HIR, low-, moderate- and high-intensity running groups.

### Tissue preparation

**Figure 1:**
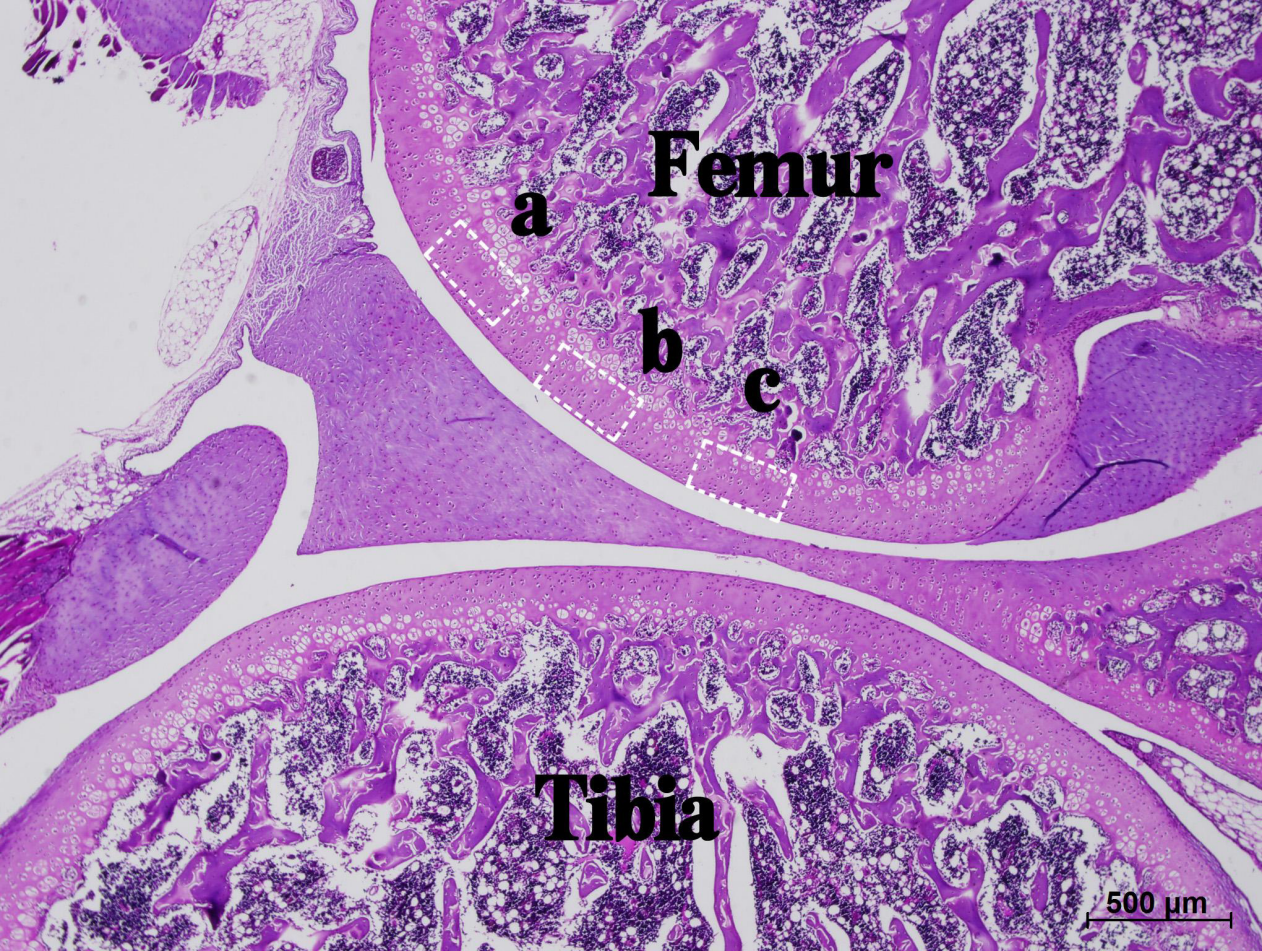
**A sagittal section of the medial midcondylar region of rat femur**. Non-contact area (**a**), transitional area (**b**), and contact area (**c**) were determined by gross observation. Scale bar = 500 μm.

For histological morphology and immunohistochemistry examinations, femoral condyles of the medial compartment on the right sides of rats in each group were dissected and fixed in 4% buffered formaldehyde pH 7.4 for 24 hours. Decalcification was completed in 10% ethylenediaminetetraacetic acid (EDTA) solution, and then the samples were embedded in paraffin wax. Thereafter, they were cut into 5-mm sagittal sections in the medial region. In each section, three areas (non-contact, transitional and contact areas) were chosen for histomorphological evaluation and immunohistological analysis (Figure 1).

For quantitative real-time PCR analysis, articular cartilage samples from femoral condyles on the left sides were obtained with a scalpel or rongeur, and flash-frozen in liquid nitrogen at -80°C.

### Histomorphological evaluation

The samples were stained with Safranin-O and histomorphologically evaluated with the modified Mankin scoring system, which has previously been applied to many experimental OA models [18,19], and proven to be sensitive to early OA changes induced by treadmill exercise [24]. All sections were graded by two independent observers (LL and YZZ) who were unaware of the grouping, and the mean score was calculated for each area in each section.

### Immunohistochemistry for lubricin

In addition to histomorphological evaluation, immunohistological analysis for lubricin was performed in the above-mentioned three areas of each section. After deparaffinization and rehydration of the tissue sections, lubricin was immunostained with the two-step immunohistochemistry method as instructed by the manufacturer.

The sections were incubated overnight with rabbit polyclonal antibody against rat lubricin (sc-98454) (Santa Cruz Biotechnology INC., Santa Cruz, CA, USA), 1:200 dilution, at 4°C. Its epitope corresponds to amino acids 1265-1404 mapping at the C-terminus of Lucricin of human origin. The slides were washed three times in PBS followed by a 30-minute incubation at room temperature with goat anti-rabbit immunoglobulin G (IgG) (Santa Cruz Biotechnology INC., Santa Cruz, CA, USA) and visualized with DAB chromagen. The slides were stained for 40 sec, and then the nucleus was counterstained with hematoxylin for 6 sec. Negative control sections were prepared using the same protocol described above, but primary antibody was replaced by PBS. All of the sections were stained concomitantly, at the same time by the same person (YZZ). For each section, three regions were digitally captured with a color video camera attached to a light microscope (Nikon H600L Microscope and image analysis system, Tokyo, Japan), at 400× magnification with constant illumination intensity. Images were captured using Image-Pro Plus software (Media Cybernetics, Silver Spring, MD, USA) with predetermined threshold parameters for lubricin staining. Areas of interest (AOI) from surfaces to the subchondral of the femoral condyle cartilage were selected using the irregular AOI tools. The IOD (integrated optical density) and area were calculated for each region using Image-Pro Plus 6.0 software. The ratio of IOD to area in each region was obtained, and subsequently averaged for lubricin content in each section.

### Quantitative real-time polymerase chain reaction (RT-PCR)

The cartilage samples were frozen in liquid nitrogen, and then were broken into pieces with a masher. Total RNA was prepared using the Trizol reagent (Invitrogen Life Technologies, Carlsbad, CA, USA) according to the manufacturer's instructions. RT-PCR was performed on 0.5 mg total RNA using a PrimeScript RT reagent kit with gDNA Eraser (Takara Biotechnology (Dalian) Co. LTD., Dalian, China). Quantitative PCR was performed using an ABI 7500 Real-Time PCR system and a QuantiTect SYBR Green PCR (Takara Biotechnology (Dalian) Co. LTD., Dalian, China); glyceraldehyde-3-phosphate dehydrogenase (*GAPDH*) was used as an endogenous reference, and each sample was normalized to its *GAPDH *content. All experiments were performed in duplicate and repeated twice. Primers for quantitative PCR are shown in Table 2.

**Table 2:** Primer sequence used in quantitative PCR

Primer	Forward	Reverse
GAPDH	5'-GGCACAGTCAAGGCTGAGAATG -3'	5'-ATGGTGGTGAAGACGCCAGTA-3'
PRG4	5'- AGGGCGTTGCATCCAAGAA -3'	5'-ACAGTTGCAGGTGGCGTCTCTA-3'
TGF-β1	5'- TGCGCCTGCAGAGATTCAAG -3'	5'- TAACGCCAGGAATTGTTGCTA-3'

GAPDH, glyceraldehyde-3-phosphate dehydrogenase; PRG4, proteoglycan; TGF-β1, transforming growth factor- β1.

### Statistical analysis

Results are expressed as the mean ± SD. Statistical analysis was carried out using one-way analysis of variance (ANOVA) and Tukey's test for post hoc analysis with significance set at *P *< 0.05. The relationship between the Mankin score versus the lubricin level was assessed with regression analysis.

## Results

### Histological observation

**Figure 2:**
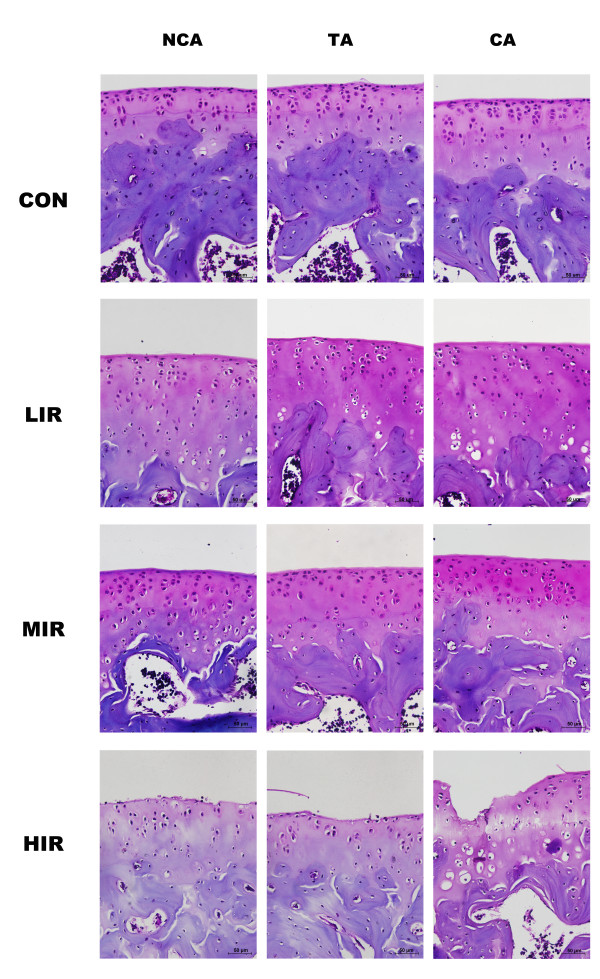
**Histological morphology of femoral articular cartilage with safranin-O staining at contact, transitional, and non-contact areas in the control (CON), low- (LIR), moderate- (MIR), and high-intensity (HIR) groups**. Grossly normal histological characteristics of cartilage sections were observed in the CON, LIR and MIR groups, though the increased staining for Safranin O was found in the LIR and MIR groups in comparison with the CON group. Within the CON, LIR, and MIR groups, no distinct difference was found among the contact, transitional, and non-contact areas. In contrast, osteoarthritic histological changes of surface irregularities, cell cloning, and marked reduction in the safranin-O staining were found in the HIR group, with the most severe changes at the contact area. NCA: non-contact area, TA: transitional area; CA: contact area. Scale bar = 50 μm.

Figure 2 shows the histological features of femoral articular cartilage with safranin-O staining at contact, transitional, and non-contact areas in the four groups. Similar to the CON group, grossly normal histological characteristics of cartilage sections were observed in the LIR and MIR groups. However, it appears that the increased staining for safranin O was in the LIR and MIR groups in comparison with CON group. Within the CON, LIR and MIR groups, no distinct differences were found among the contact, transitional, and non-contact areas. In contrast, osteoarthritic histological changes of surface irregularities, cell cloning, and marked reduction in the safranin-O staining were found in the HIR group, with the most severe changes at the contact area.

**Figure 3:**
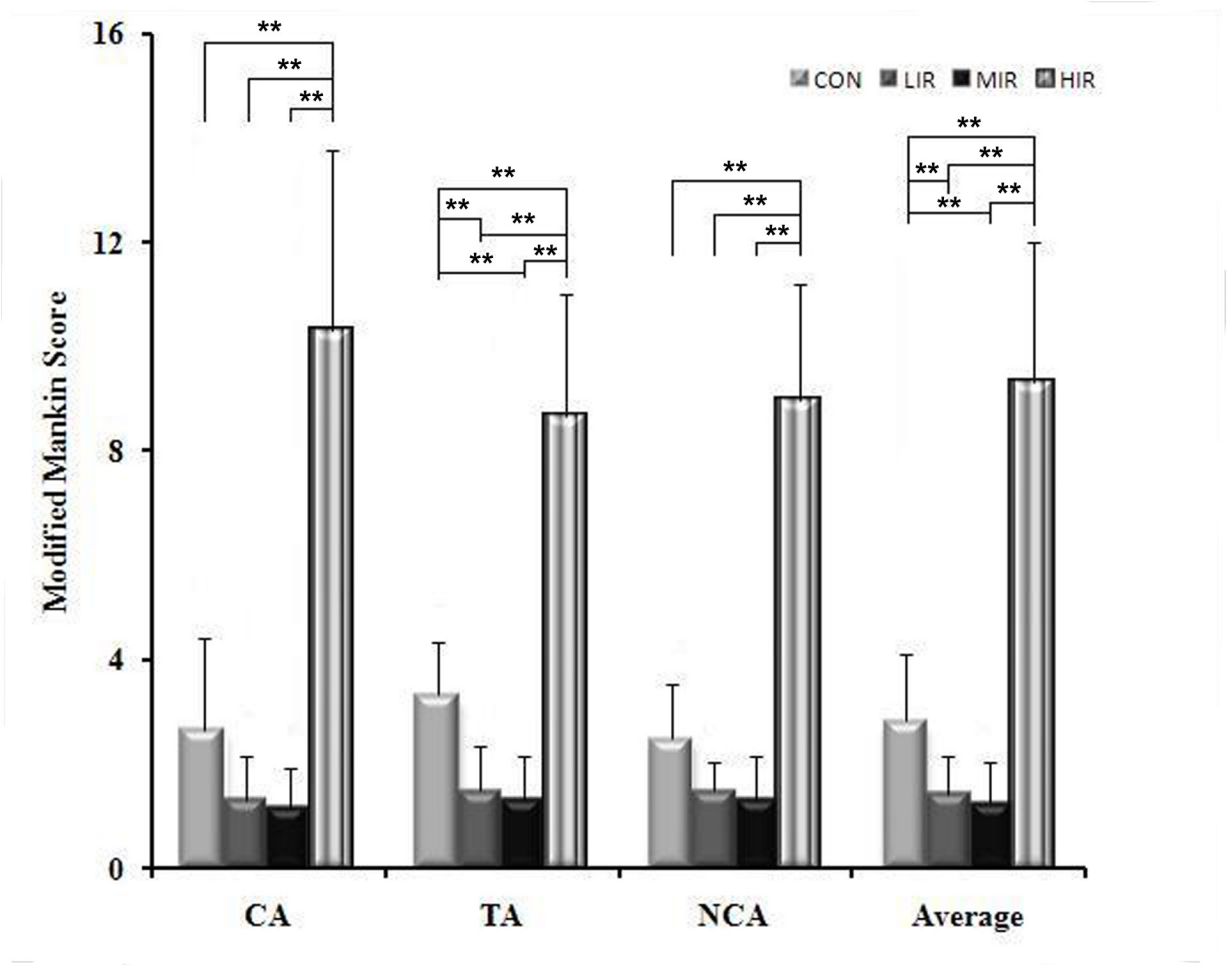
**Mankin score at the contact, transitional, and non-contact areas and average for all three areas in the control (CON), low- (LIR), moderate- (MIR), and high-intensity (HIR) groups**. Similar changes were observed for each area as well as the average: both the LIR and MIR groups had a lower Mankin score than the CON group, and a significantly higher score was found in the HIR compared to the CON, LIR, and MIR groups, respectively. No statistical difference was found between the LIR and MIR groups. NCA: non-contact area, TA: transitional area; CA: contact area.

Histomorphological evaluation was made by the modified Mankin scoring system at contact, transitional, and non-contact areas in the four groups. A similar pattern of changes was observed for each area as well as for the average (Figure 3): both the LIR and MIR groups had lower Mankin score than the CON group. However, a significantly higher Mankin score was found in the HIR group compared to the CON, LIR, or MIR groups, respectively. No statistical difference was found between the LIR and MIR group.

### Immunohistochemistry of lubricin

**Figure 4:**
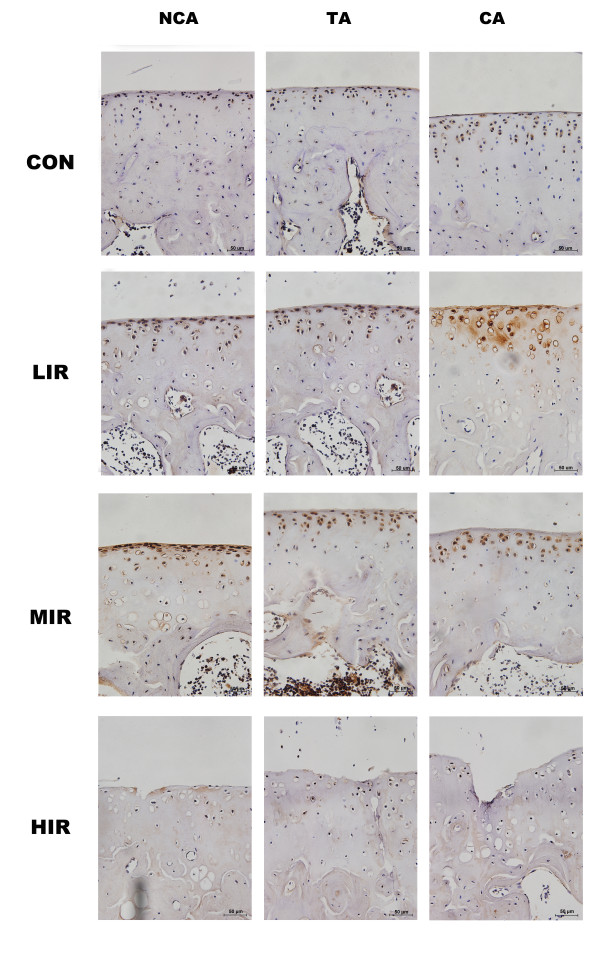
**Immunostaining of lubricin of femoral articular cartilage at contact, transitional, and non-contact areas in the control (CON), low- (LIR), moderate- (MIR), and high-intensity (HIR) groups**. Lubricin was mainly immunolocalised to chondrocytes and extracellular matrix (ECM) in the superficial zone of normal cartilage. Markedly increased staining of lubricin was found in the LIR and MIR groups, whereas little staining was detected in the HIR group. NCA: non-contact area, TA: transitional area; CA: contact area. Scale bar = 50 μm.

Figure 4 shows the immunostaining of lubricin in cartilage sections at the three areas in the four groups. Lubricin was mainly immunolocalised to chondrocytes and extracellular matrix (ECM) in the superficial zone of normal cartilage. Markedly increased staining of lubricin was found in the LIR and MIR groups, whereas little staining was detected in the HIR group.

**Figure 5:**
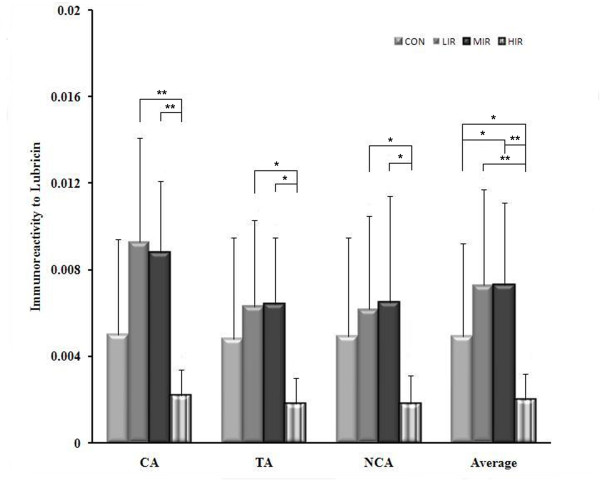
**Lubricin content at the contact, transitional, and non-contact areas and average for all three areas in the control (CON), low- (LIR), moderate- (MIR), and high-intensity (HIR) groups**. Similar changes were observed for each area as well as the average: both the LIR and MIR groups had higher lubricin content than the CON group, and significantly lower lubricin content was found in the HIR group compared to the LIR and MIR groups. No statistical difference was found between the LIR and MIR groups. NCA: non-contact area, TA: transitional area; CA: contact area.

Figure 5 indicates the lubricin content at the contact, transitional, and non-contact areas in the four groups. For each group, the lubricin content for the three areas was averaged. Among the four groups, a similar pattern of changes was observed for area as well as the average. Both the LIR and MIR groups had higher lubricin content than the CON group with statistical significance only for the average. However, significantly lower lubricin content was found in the HIR group, compared to the LIR and MIR groups at the three areas. On average, a statistically significant difference was found between the CON and HIR groups. No statistical difference was found between the LIR and MIR groups. In addition, a significant inverse correlation (*P *< 0.01) was found between the lubricin content and the Mankin score either in each group or as a whole (Table 3).

**Table 3:** Correlation between lubricin content and Mankin score

Group				
	
CON (*n *= 18)	LIR (*n *= 18)	MIR (*n *= 18)	HIR (*n *= 18)	Total (*n *= 72)
0.593**	0.692**	0.76**	0.816**	0.42**

CON, control group; LIR, MIR and HIR, low-, moderate- and high-intensity running groups. **Significantly different from zero *P *< 0.01.

### Real-time quantitative PCR for *PRG4 *and transforming growth factor (*TGF*)-β

**Figure 6:**
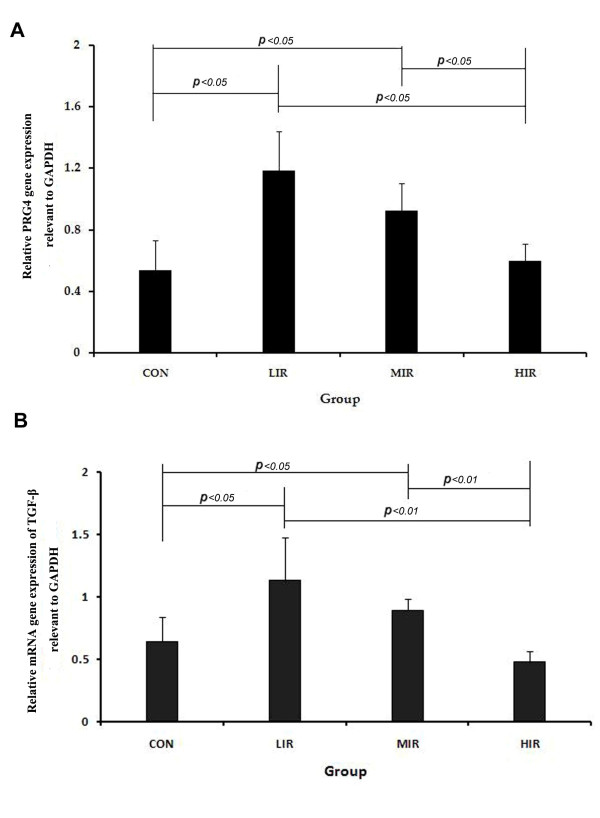
**Relative mRNA gene expression of proteoglycan (*PRG*)4 (A) and transforming growth factor (*TGF*)-β (B) relevant to lyceraldehydes-3-phosphate dehydrogenase (GAPDH) in the control (CON), low- (LIR), moderate- (MIR), and high-intensity (HIR) groups**. Significantly higher mRNA gene expression of *PRG*4 was found in the LIR (1.03 ± 0.38) and MIR group (1.00 ± 0.12) than in the CON group (0.67 ± 0.31). However, there was significantly lower *PRG*4 expression in the HIR group (0.64 ± 0.09) than in LIR or MIR groups. Similar changes were found in mRNA gene expression of *TGF*-β. The LIR (1.13 ± 0.35) and MIR (0.89 ± 0.09) groups had significantly higher gene expression than the CON group (0.65 ± 0.20), whereas the HIR group (0.48 ± 0.08) had significantly lower expression than the LIR or MIR groups. NCA: non-contact area, TA: transitional area; CA: contact area.

As shown in Figure 6A, there was a statistically significant increase of mRNA gene expression of *PRG4 *in the LIR (1.03 ± 0.38) and MIR groups (1.00 ± 0.12) than in the CON group (0.67 ± 0.31). However, *PRG4 *expression was significantly lower in the HIR group (0.64 ± 0.09) than that in the LIR or MIR groups. A similar pattern of changes was found in mRNA gene expression of *TGF*-β. The LIR (1.13 ± 0.35) and MIR (0.89 ± 0.09) groups had significantly higher gene expression than the CON group (0.65 ± 0.20), whereas, the HIR group had significantly lower expression (0.48 ± 0.08) than the LIR or MIR groups (Figure 6B).

## Discussion

It is well recognized that exercise and joint loading can alter articular cartilage composition through alterations in chondrocyte metabolism [25]. Nevertheless, their effects on cartilage lubricin, a mucinous glycoprotein synthesized by chondrocytes in the superficial zone of articular cartilage [1], remain poorly understood. To our knowledge this is the first report describing the immunolocalisation and gene expression of cartilage in response to treadmill running. Our results suggest a marked intensity-specific effect of running on the immunolocalisation and gene expression of lubricin in cartilage. In addition, a similar running-intensity-dependent effect was found on the gene expression of *TGF*-β.

Previously, biomechanical regulation of metabolism of lubricin by chondrocytes was well documented *in vitro *[11-14]. As suggested by the current study, running-induced joint loading has significant effects on the *in vivo *synthesis of lubricin, and such effects vary with running intensity. What is more, a significant inverse correlation between the lubricin level and Mankin score is demonstrated, indicating that lubricin might be partially responsible for the change in cartilage. Low- or moderate-intensity running is associated with up-regulation of *PRG4*, leading to the increase in secretion and synthesis of lubricin. It is well accepted that lubricin plays an important role in articular joint physiology, because it can provide an important property for normal joint function by conferring wear resistance in addition to boundary lubrication. Therefore, low- to moderate-intensity running should have beneficial effect on the health of normal cartilage by up-regulating the biosynthesis of lubricin. On the other hand, exercise is one of the most widely prescribed nonpharmacological therapies for OA management. However, the mechanism underlying its benefit is largely unknown [26]. Galois *et al. *[27] reported that moderate-intensity running can decrease severity of experimental OA in rats. Although further investigations are needed, it is supposed that appropriate exercise can protect against increased cartilage damage by increasing the secretion and synthesis of lubricin in degenerative cartilage. Substantial evidence has been provided on the role of intra-articular lubricin administration in the prevention of post-traumatic OA [11,28-30].

Conversely, our results showed that excessive running-induced load leads to decreased lubricin level and gene expression. Recently, Elsaid *et al. *[30] reported that forced joint exercise resulted in decreased lubricin cartilage expression, increased cartilage degeneration and reduced superficial zone chondrocyte viability in the ACLT (anterior cruciate ligament transaction) joint. Using a meniscectomy-induced (that is, mechanically induced) OA model in sheep, Young *et al. *[9] reported that abnormal joint motion resulted in degeneration of articular cartilage in certain regions of the tibial plateau, where lubricin gene expression decreased. A number of previous studies, including ours [19-21,24], have shown that excessive load has a detrimental effect on cartilage. Such detrimental effect is quite likely to be associated with the decrease in lubricin level due to the significant inverse correlation between the lubricin level and Mankin score indicated in the current study. Coles *et al. *[31] found that the deletion of lubricin resulted in significant structural and biomechanical changes in the articular cartilage with age, again, suggesting a significant role of lubricin in preserving normal joint structure and function. A recent study showed that a mouse with only one functional allele for lubricin did not provide chondroprotection *ex vivo *as its joint was loaded and oscillated [32], which is consistent with our finding that, under condition of excessive wear, lubricin as a sacrificial boundary layer may succumb to overuse. Taken together, it is believed that the down-regulation of lubricin by excessive running load, at least partially, contributes to the cartilage damage.

Similarly to previous reports of lubricin immunolocalisation in normal cartilage [5,9], lubricin-positive cells and matrix were observed mainly in the superficial zone in the current study. However, site-associated variation was detected between contact, non-contact, and transitional regions. Similar results were previously reported by Nugent-Derfus *et al. *[14]. Such variation was thought to be associated with the apparent dependence on the local mechanical environment in different regions over the surface of the cartilage [33-35]. Taken together with the altered lubricin level and gene expression patterns subjected to different running-induced loads, this investigation further supports the hypothesis that mechanical factors are key determinants of lubricin metabolism *in vivo*.

Interestingly, under different mechanical conditions (that is, low-, moderate-, and high-intensity running load), a synchronous changing pattern was found for lubricin levels in three different areas of the cartilage surface. For each area, the lubricin level increased under a low- or moderate-running-induced load, and decreased under excessive running load. This implies a corresponding safe upper limit of stress for each area. As the corresponding stress acting on different areas differs, the stress threshold should not be identical in these areas. According to the so-called conditioning hypothesis proposed by Seedhom [36], the stress threshold of cartilage is regulated by the prevalent stresses arising in joints. We therefore postulate that the contact region has a higher stress threshold than the other regions due to its relatively higher prevalent stress.

The mechanisms involved in regulating lubricin gene expression and synthesis remain largely unknown. In the current study, similar to *PRG*4, the expression of *TGF*-β was up-regulated under a low- and moderate-running load, but down-regulated under excessive running load. Neu *et al. *[35] suggested that mechanotransduction of lubricin expression in articular cartilage occurred through *TGF*-β-mediated signaling pathways. A body of evidence has showed that *TGF*-β stimulates lubricin synthesis [37-40], and thus may be beneficial for normal cartilage function [2,41]. It is therefore assumed that the regulation of running-induced load on the biosynthesis of lubricin is through the *TGF*-β mediated signaling pathway, or a combination with the direct mechanical regulation. Future work will be necessary to understand the precise mechanisms.

## Conclusions

In the current study, a marked intensity-specific effect of running on the immunolocalisation and gene expression of lubricin in cartilage is demonstrated. In addition, the significant inverse correlation between the lubricin level and the Mankin score indicates that lubricin might be partially responsible for the change in cartilage. Our findings provide evidence that mechanical factors are key determinants of lubricin metabolism *in vivo*.

## Abbreviations

ACL: anterior cruciate ligament; ANOVA: analysis of variance; AOI: areas of interest; CPM: continuous passive motion; ECM: extracellular matrix; HIR: high intensity running; IgG: immunoglobulin G; IOD: integrated optical density; LIR: low intensity running; MIR: moderate intensity running; OA: osteoarthritis; PBS: phosphate-buffered saline; RT-PCR: real-time polymerase chain reaction; PRG4: proteoglycan 4; SZP: superficial zone protein; TGF: transforming growth factor.

## Competing interests

The authors declare that they have no competing interests.

## Authors' contributions

GN conceived the study, participated in the design, and wrote most of the manuscript. LL and YZ performed the experiments, analyzed data and helped to draft the manuscript. All authors read and approved the final manuscript.
